# Draft Genome Sequence of Mycobacterium porcinum CSURP1564

**DOI:** 10.1128/genomeA.00291-18

**Published:** 2018-04-19

**Authors:** A. Bouam, A. Levasseur, M. Drancourt

**Affiliations:** a Aix Marseille Université, IRD, APHM, MEPHI, IHU–Méditerranée Infection, Marseille, France

## Abstract

Mycobacterium porcinum is a rapidly growing environmental mycobacterium responsible for opportunistic infections. The 7,025,616-bp draft genome of M. porcinum strain CSURP1564 exhibits a 66.71% G+C content, 6,687 protein-coding genes, and 65 predicted RNA genes. *In silico* DNA-DNA hybridization confirms its assignment to the Mycobacterium fortuitum complex.

## GENOME ANNOUNCEMENT

Mycobacterium porcinum, a member of the Mycobacterium fortuitum third biovariant complex, is a nontuberculous mycobacterium primarily recovered from swine with lymphadenitis (1). M. porcinum is indistinguishable from the closely related Mycobacterium conceptionense on the sole basis of 16S rRNA gene sequence analysis but could be identified by partial *rpoB* gene sequencing (2, 3). M. porcinum has been detected in various environments, including drinking water (4, 5), fish, and fresh vegetables for human consumption (6, 7). M. porcinum is known to infect wild and domestic animals (8) and exhibits a zoonotic potential, being isolated from bovine milk (9). Accordingly, M. porcinum is an opportunistic pathogen responsible for wound infections (10), respiratory tract infections (11–14), bacteremia related to blood catheters (10) and peritonitis complicating dialysis catheter infections (15), and postoperative infections (16, 17).

M. porcinum CSURP1564 was cultured on Middlebrook 7H11 agar supplemented with 10% (vol/vol) oleic acid-albumin-dextrose-catalase (Becton, Dickinson, Sparks, USA) under a 5% CO_2_ atmosphere. Colonies were confirmed by matrix-assisted laser desorption ionization–time of flight mass spectrometry (18). Reads issued from sequencing genomic DNA by MiSeq technology (Illumina, Inc., San Diego, CA, USA) were assembled using SPAdes (19), and contigs were combined by using SSPACE (20), GapFiller (21), and manual finishing. This analysis yielded 8 scaffolds and 23 contigs. Then, genomic DNA was sequenced using the MinION device and an SQK-LSK108 kit (Oxford Nanopore, Oxford, UK) after purification using AMPure XP beads (Beckman Coulter, Inc., Fullerton, CA, USA). The library was quantified by a Qubit assay with the high-sensitivity kit (Life Technologies, Carlsbad, CA, USA) at 27.5 ng/µL; 842 active pores were detected for the sequencing, and the WIMP workflow was chosen for bioinformatic real-time analysis, leading to 54,579 analyzed reads after 23.5 h of sequencing. Adding MinION reads to MiSeq reads yielded five contigs assembled into two scaffolds with 7,025,616 bp and a G+C content of 66.71%. Annotation using Prokka version 1.12 (22) yielded 6,752 predicted genes, 6,687 protein-coding genes, and 65 RNA genes, including 58 tRNAs, 3 rRNA operons, and 1 transfer-messenger RNA. The M. porcinum CSURP1564 genome was incorporated into *in silico* DNA-DNA hybridization (DDH) (23) using GGDC version 2.0 (24), with reference genomes selected on the basis of the 16S rRNA gene sequence. This yielded 99.7% sequence similarity with M. porcinum IP141460001 (GenBank accession number MVIG00000000), 49.5% with M. boenickei CIP 107829 (FUWC00000000), 34.5% with M. conceptionense D16 (CTEF00000000), 34.4% with M. farcinogenes DSM 43637 (CCAY000000000) and M. neworleansense ATCC 49404 (CWKH00000000), 31.40% with M. fortuitum CT6 (NCBI reference sequence number NZ_CP011269), and 20.5% with M. avium 104 (NCBI reference sequence NC_008595).

This result confirmed that M. porcinum strain CSURP1564 differs from M. conceptionense in the M. fortuitum complex. This is the first report of MinION technology applied to the genome sequencing of a nontuberculous Mycobacterium species (25). In our experience, MinION technology has been helpful in determining genome backbones. Reporting on the M. porcinum CSURP1564 genome sequence will help to establish DNA-based methods for its detection and identification in environmental, animal, and clinical specimens to complement the *rpoB* gene that we previously developed (2, 26).

### Accession number(s).

The draft genome sequence of M. porcinum CSURP1564 has been deposited at the European Bioinformatics Institute (EBI), European Nucleotide Archive (ENA), under the accession number OLMG00000000 (OLMG01000001 to OLMG01000005).
